# Comparison of the removal of intracanal medicaments used in regenerative endodontics from root canal system using needle, ultrasonic, sonic, and laser-activated irrigation systems

**DOI:** 10.1007/s10103-024-03980-w

**Published:** 2024-01-12

**Authors:** Sıla Nur Usta, Berat Akın Erdem, Mustafa Gündoğar

**Affiliations:** 1 Department of Endodontics, Gulhane Faculty of Dentistry, University of Health Sciences, Ankara, Turkey; 2 Department of Endodontics, Faculty of Dentistry, University of Medipol, Istanbul, Turkey

**Keywords:** Calcium hydroxide, EDDY, Endodontics, Passive ultrasonic irrigation, SWEEPS

## Abstract

This study aimed to compare the syringe-needle irrigation (SNI), passive ultrasonic irrigation (PUI), EDDY, and shock wave–enhanced emission photoacoustic streaming (SWEEPS) techniques regarding calcium hydroxide and double antibiotic paste removal from the root canal in regenerative endodontic treatments. Eighty single-rooted human teeth were decoronated and enlarged up to #100 to stimulate the immature tooth model. Root canals were irrigated with 1.5% sodium hypochlorite followed by saline solution according to the regenerative endodontic treatment protocol. Dressed teeth were divided into 2 main groups regarding the used intracanal medicaments. Calcium hydroxide and double antibiotic paste were introduced to the canals, and teeth were stored for 3 weeks. Each medicament group was divided into 4 subgroups according to the activation techniques. Medicaments were removed using a 17% EDTA solution. Teeth were split longitudinally into two parts. The remaining medicaments were evaluated under a stereo microscope with a scoring system. Data were analyzed with the Kruskal-Wallis and Mann-Whitney *U* tests. Regardless of the used irrigation activation systems, there was no statistically significant difference between the removal of the CH and DAP from the root canal (*P*>0.05). While SWEEPS had the highest ability regarding the removal of intracanal medicaments, syringe-needle irrigation had the lowest (*P*<0.05). There was no statistically significant difference between PUI and EDDY (*P*>0.05). Complete removal of intracanal medicaments could not be achieved with any techniques. SWEEPS technology was more effective in removing intracanal medicaments in regenerative endodontic treatments compared to the sonic and ultrasonic irrigation activation systems.

## Introduction

Regenerative endodontic treatments (RETs) are considered biologically based procedures designed to regenerate and replace damaged structures, including the pulp-dentin complex in necrotic immature teeth following dental caries or trauma [1]. According to the guideline of the American Association of Endodontists (AAE) and European Society of Endodontology (ESE) [2, 3], RETs include disinfection of the root canal space, the invocation of the stem cells and growth factors, and the generation of a scaffold that promotes the new tissue by creating a blood clot. Crucial steps for regenerative procedures are root canal disinfection and preventing reinfection. Due to the short and open apexes and thin dentin walls of the necrotic immature teeth, mechanical instrumentation is not suggested, and the absence of preparation may result in unremovable bacterial biofilm [4]. Therefore, it is essential to use effective irrigation solutions and intracanal medicaments that have superior antibacterial properties to ensure a suitable environment for regeneration through the removal of biofilms [5].

Triple antibiotic paste (TAP) that contains equal portions of metronidazole, ciprofloxacin, and minocycline; double antibiotic paste (DAP) obtained by removing minocycline from TAP; and calcium hydroxide (CH) are the most widely used medicaments for root canal disinfection [6]. Although using these medicaments is essential to provide bacteria-free root canal space, they have been associated with some detrimental effects such as being cytotoxic [7], causing discoloration [8], resulting in bacterial resistance [9], and reducing the bond strength of barrier materials to the dentin [6]. Therefore, the complete removal of intracanal medicaments from root canal walls is one of the important factors for the long-term success of RETs [8].

Intracanal medicaments can be removed with the aid of irrigation solutions and the action of mechanical instrumentation [10]. However, the absence of mechanical instrumentation in RETs might jeopardize the removal of intracanal medicaments and consequently prevent the formation of desirable conditions for stem cell survival, proliferation, and differentiation. Based on present guidelines, intracanal medicaments are removed by syringe-needle irrigation (SNI) with ethylene diamine tetra acetic acid (EDTA) solution in routine regenerative treatments [2, 3]. However, since SNI could compromise the effective delivery of solution into the root canal space, various irrigation activation systems have been introduced to improve agitation [11]. One of these activation systems, passive ultrasonic irrigation (PUI), relies on the transmission of acoustic energy from an oscillating file or smooth wire to an irrigation solution in the root canal and can be activated from 25 to 30 kHz [12]. EDDY (VDW, Munich, Germany) is another irrigation activation system that is used in endodontic treatments by application of sonic energy. EDDY has a flexible polyamide tip that triggers cavitation and acoustic streaming in the irrigation solution and can be operated between 5 and 6 kHz [13]. It was shown that both PUI and EDDY are effective systems for removing intracanal medicaments in routine endodontic treatments [10, 14].

Laser-activated irrigation has been developed to improve the irrigation and disinfecting efficacy of irrigation solutions. One of the latest laser technology in endodontics, photon-induced photoacoustic streaming (PIPS, FOTONA, Ljubljana, Slovenia, EU), uses the Er:YAG laser to ensure a three-dimensional (3D) flow of the irrigation solution within root canal space and, subsequently, provides advance disinfection by creating cavitation and photoacoustic shock waves [15]. In recent years, shock wave–enhanced mission photo-acoustic streaming (SWEEPS, FOTONA, Ljubljana, Slovenia, EU) lasers have been introduced in endodontics to increase the debridement efficiency of the PIPS [16]. SWEEPS technology uses the erbium laser with ultra-short pulses for collapsing of laser-induced bubbles by placing its fiber tip in the pulp chamber similar to the PIPS [17]. As the initially formed bubble collapse, energy is emitted to form the secondary bubble, and expansion of the secondary bubble accelerates the collapse of the primary bubble, leading to advanced shockwave emission even inside the narrowest root canals [15].

Although the efficacy of SWEEPS technology in removing intracanal medicaments has been demonstrated in the literature [18, 19], its effectiveness in RETs without mechanical preparation has not been investigated and compared with currently used irrigation activation systems. Therefore, the aim of this study is to evaluate the removal of DAP and CH from the canal space using SNI, PUI, EDDY, and SWEEPS techniques. The null hypothesis was the different irrigation activation systems used in RETs would not differ regarding the removal of intracanal medicaments from the root canal space.

## Materials and methods

### Sample selection, preparation, and placement of intracanal medicaments

The study design (no. 2023-144) was approved by the Research Ethics Committee of the University. The required sample size was calculated using G*Power software (G*Power 3.1.9.4, Heinrich-Heine, Dusseldorf, Germany) and 10 teeth per group were allowed for comparison of the quantitative variables between groups at alpha error probability of 0.05 and power of 0.80 [6]. Teeth with caries-free, single-rooted, and closed apex were collected and evaluated under a stereo microscope for any possible fractures or anatomical malformations. Accordingly, the periodontal tissues of selected 80 teeth were removed from the external root surfaces with periodontal curettes, and teeth were stored in 0.1% thymol solution at 4°C until used.

The immature teeth models were created based on a similar study in the literature [20]. Following 3-mm resection of the apical ends, the root lengths were adjusted to 14±1 mm. Since the critical apical diameter in RETs is 1.1 mm, the root canal spaces of selected teeth were enlarged up to #100 (Dentsply Tulsa) to obtain a standard intracanal diameter [21]. Between each file, irrigation procedures were performed with SNI using a sterile saline solution. Subsequently, the root canals were irrigated with 20 mL 1.5% sodium hypochlorite (NaOCl) for 5 min in accordance with regenerative protocols proposed by AAE and ESE [2, 3], rinsed with 20 mL saline, and dried with paper points. The apical ends of roots were sealed with modeling wax and vacuum suction was used during irrigation procedures to mimic clinical conditions. Afterwards, teeth were divided into 2 main groups based on the type of intracanal medicaments. Intracanal medicaments were prepared as the manufacturer’s recommendations and applied using lentulo spiral (VDW, Munich, Germany). The modeling waxes were removed to ensure that intracanal medicaments could be placed in the whole root canal lumen through the apical foramen. Preparations were performed as described below:

DAP (*n* = 40): Equal portions of metronidazole (Flagyl, Sanofi, Istanbul, Turkey) and ciprofloxacin (Cipro, Biofarma, Istanbul, Turkey) were mixed with distilled water to a final concentration of 1–5 mg/mL.

CH (*n* = 40): CH powder (Kalsin, Aktu Tic, Izmir, Turkey) was mixed with distilled water at a 1:1 ratio.

After observing the intracanal medicaments that had been placed properly, the modeling waxes were re-placed, and access cavities were restored with a temporary filling material (Cavitimi, Imicryl Dental, Turkey). Teeth were stored at 37°C in %95 relative humidity for 3 weeks to simulate RET protocol.

### Removal of intracanal medicaments

After 3 weeks, all dressed teeth in DAP and CH groups were randomly divided into four subgroups (*n*=10) in terms of the irrigation activation system and irrigated with 20 mL 17% EDTA and saline solution based on present guidelines by ESE, respectively [3]. While EDTA solution can significantly increase the release of growth factors, which promote stem cell differentiation from the dentine matrix, the saline solution was also recommended in order to reduce possible adverse effects of irrigants on target cells [3]. All irrigation procedures were performed by the same operator in the same conditions to ensure standardization. Additionally, the irrigation process was completed with modeling waxes to mimic clinical conditions, and waxes were kept until teeth were longitudinally sectioned. Used irrigation systems were activated according to the manufacturer’s recommendations as follows:

SNI: The tip of a 30-gauge side-vented closed-ended needle (Endo-Top, Cerkamed, Stalowa Wola, Poland) was placed 1 mm short of the root apex and moved in an up-and-down motion. Each 5 mL solution was delivered into the root canal for 20 s.

PUI: A stainless steel (#25) file (Irri-Safe; Acteon, Merignac, France) driven by an ultrasonic device (Suprasson PMax de Satelec Acteon, Merignac, France) was placed 1 mm short of the root apex and every 5 mL solution was activated for 20 s.

EDDY: A non-cutting polyamide tip (#25) operated by an air-driven handpiece (Kavo Kerr, Detroit, USA) was placed 1 mm short of the root apex and each 5 mL solution was activated for 20 s at maximum intensity.

SWEEPS: Er:YAG laser with a wavelength of 2940 nm and a pulse length of 50 μs equipped with the handpiece H14 was adjusted using a special mode for SWEEPS (LightWalker AT, Fotona, Ljubljana, Slovenia; 0.3 W, 15 Hz, and 20 mJ, without water or air). The 9-mm-long and tapered 600-μm fiber tip (PIPS 600/9) was placed in the access cavity and activated at a fixed position during the procedures. Every 5 mL solution was activated for 20 s.

### Evaluation of the remaining intracanal medicaments

**Fig. 1:**
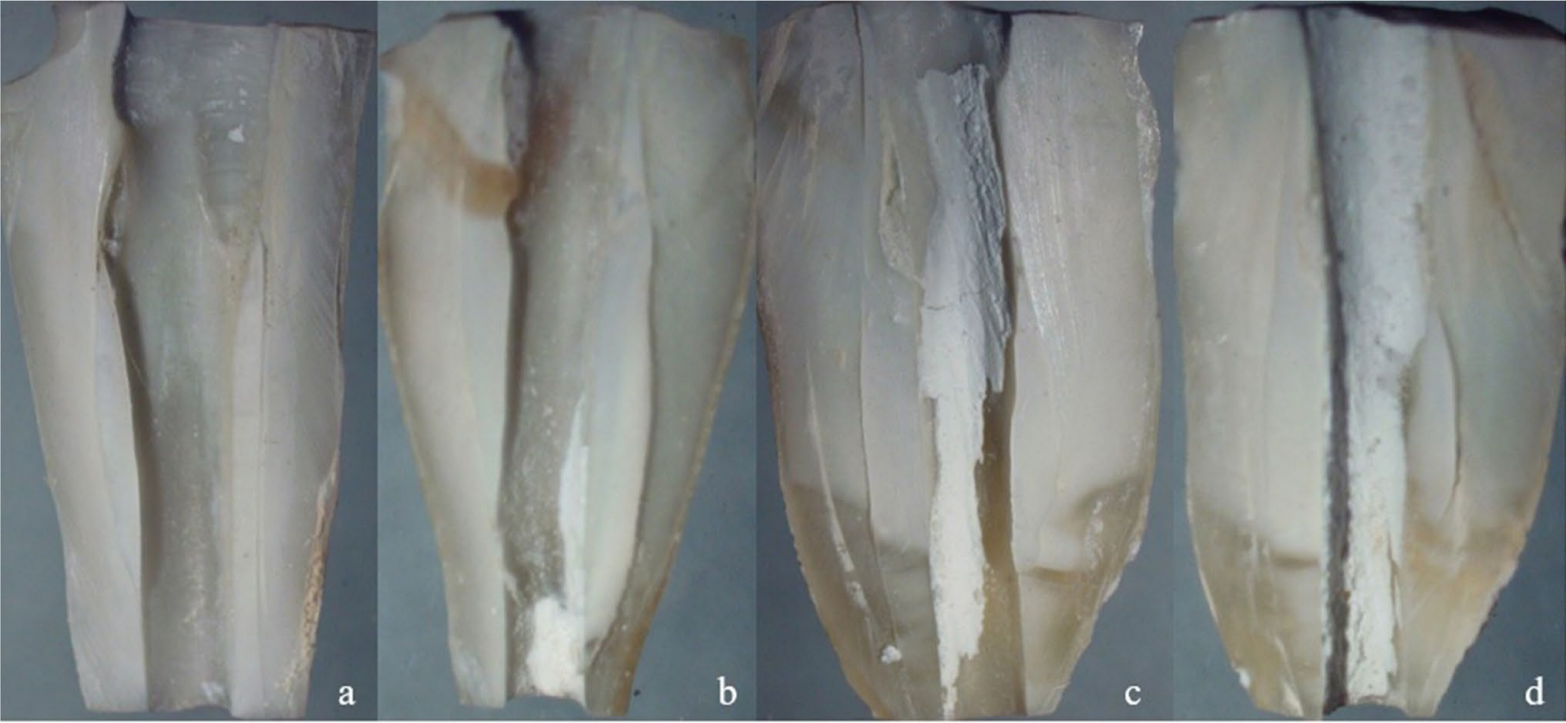
Demonstrative images of score 0 (**a**), score 1 (**b**), score 2 (**c**), and score 3 (**d**)

Following the irrigation procedures, modeling waxes were removed and teeth were longitudinally sectioned into two halves using a diamond disk. During this procedure, special care was taken to avoid penetration of the disk in the root canal system. After obtaining enough space for the separation of the teeth, an enamel chisel was inserted in the grooves, and a smooth pressure was applied to separate the two parts [22]. Root samples were examined under a stereo microscope (Olympus SZ61, Olympus, Tokyo, Japan) at a 20× magnification, and images were taken with a digital camera (Olympus DP12, Olympus, Tokyo, Japan) connected to the microscope. Each image was evaluated using the Image J software program (Image J 1.47V, National Institute of Health, USA). Firstly, the border of the whole root canal space was determined and measured and this measurement corresponded to 100%. Subsequently, the area of the remaining intracanal medicament was measured and these two measurements were proportioned to each other. According to the obtained results as a percentage, each value was also independently scored by 2 calibrated and blinded investigators according to the scoring system that has been proposed in the literature previously [23]. The used scores were as follows: 0 = empty cavity, 1 = <50% of the cavity is filled with intracanal medicaments, 2 = >50% of the cavity is filled with intracanal medicaments, and 3= the cavity is completely filled with intracanal medicaments (Fig. 1). Scores of the selected 40 samples (25%) were re-evaluated after 1 week to ensure accuracy.

### Statistical analysis

Statistical Package for Social Sciences software (SPSS 26, Chicago, IL, USA) was used for statistical analysis. The Cohen kappa test was used to analyze the inter-examiner agreement. The Shapiro-Wilk test served to check the normality of the variables. The Kruskal-Wallis test and Mann-Whitney *U* test with Bonferroni correction were used to compare the remnant of CH and DAP within different medicament groups. The Mann-Whitney *U* test was used to compare two independent groups. The significance level was 5%.

## Results

**Fig. 2:**
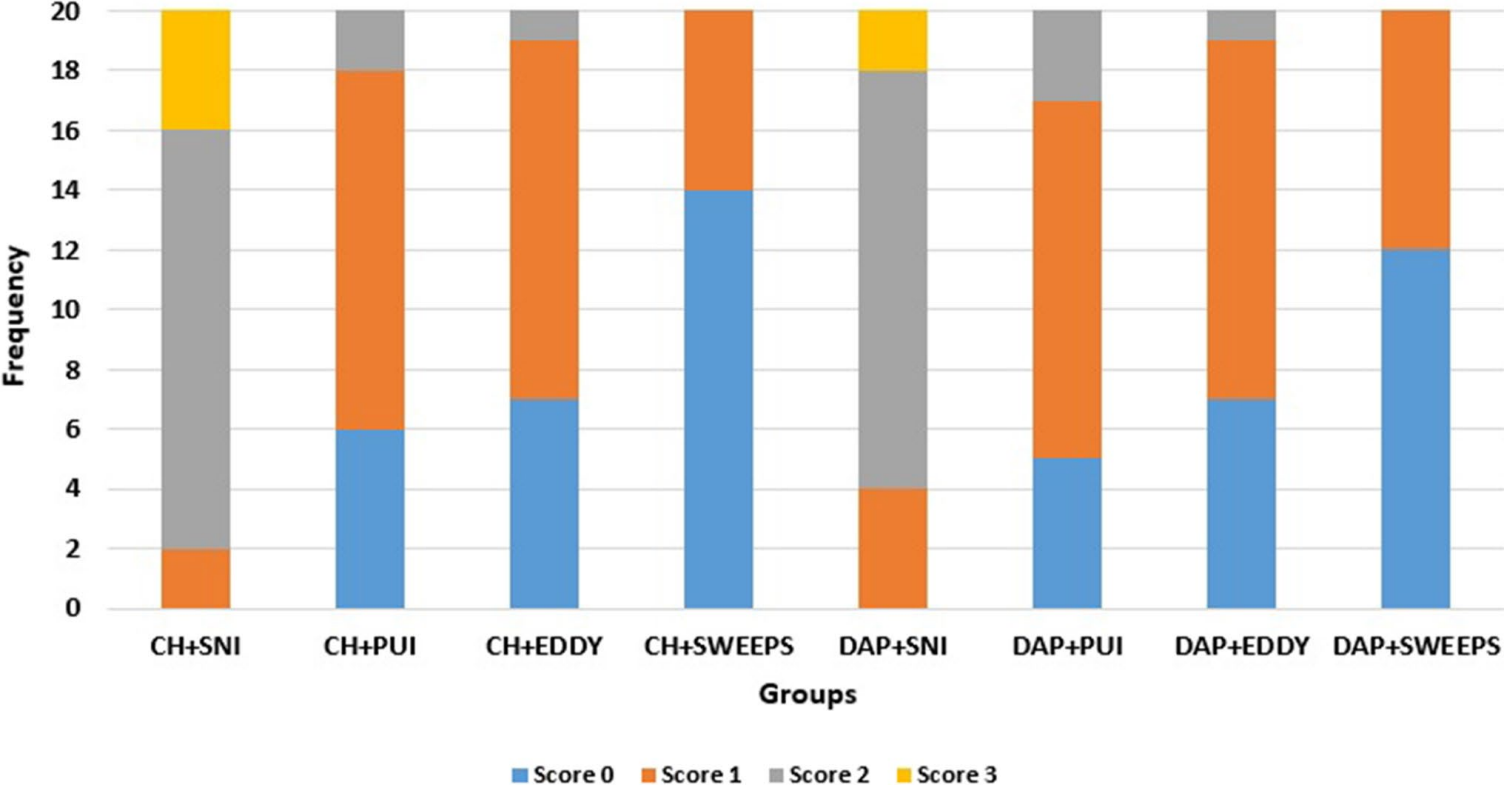
The distributions of the scores based on the experimental groups

The Cohen kappa value was calculated as 0.921 for inter-examiner agreement. Table 1 shows the percentages of the remaining intracanal medicaments based on different irrigation activation systems. The distribution of the scores for each group is also demonstrated in Fig. 2.

**Table 1:** The percentages of the remaining intracanal medicaments for four different irrigation activation systems.

	SNI	PUI	EDDY	SWEEPS
CH	66.17^a,1^	31.54^b,1^	29.32^b,1^	11.74^c,1^
DAP	64.33^a,1^	27.67^b,1^	25.04^b,1^	12.84^c,1^

Different superscript lowercase letters in the same row indicate a statistically significant difference (*P* < 0.05)

The same superscript numbers in the same column indicate no statistically significant difference (*P* > 0.05)

Regardless of the used irrigation activation systems, there was no statistically significant difference between the removal of the CH and DAP from the root canal system (*P* > 0.05). Moreover, while the efficiency of SWEEPS regarding the removal of both used intracanal medicaments from the root canal system was significantly higher (*P* < 0.05), the SNI group showed the lowest effectiveness compared to the other used systems (*P* < 0.05). No statistically significant difference was found between PUI and EDDY systems in terms of intracanal medicament removal (*P* > 0.05).

Within each type of intracanal medicament group, SWEEPS also removed the highest amount of medicaments and SNI removed the least (*P* < 0.05). PUI and EDDY groups indicated similar effectiveness regarding the medicament removal (*P* > 0.05).

## Discussion

It has been showed that overall success rates for the regenerative endodontic treatments ranged from 50 to 98% and the survival rates were between 94% and 100% through development of used materials and techniques [24]. Especially using intracanal medicaments to eliminate microorganisms is the crucial part of regenerative treatments in order to achieve enhanced disinfection and increased the success rates [25]. However, due to the some adverse effects of currently used intracanal medicaments on the viability of stem cells and the bond strength of the barrier materials, their application in RETs presents limitations [26]. Therefore, based on the knowledge that the complete removal of intracanal medicaments from the root canal system is essential [6, 8], this study aimed to compare the effect of different irrigation activation methods on the removal of CH and DAP in RETs. The null hypothesis was rejected since SWEEPS removed significantly higher amounts of intracanal medicaments compared to the other used systems.

The remaining intracanal medicaments in the root canal system have been evaluated with several techniques in previous studies such as digital photographs [27], stereomicroscopy [6], scanning electron microscopy [28], and micro-computed tomography (micro CT) [29]. This study assessed the amount of the remaining medicament in the root canal space with a 4-grade scoring system by evaluating the images under a stereo microscope at a 20× magnification [23] since the scoring system has several advantages such as ease of application and repeatability and high rates of intra-examiner agreement used [30]. On the other hand, it has been stated that measuring the surface layer of intracanal medicaments poses a risk in demonstrating the three-dimensional (3D) evaluation of the removal depth [31], and therefore volumetric analysis with micro-CT could demonstrate more accurate results. However, three-dimensional imaging with micro-CT has low availability and high cost [31]. In addition, the radiopacity level of CH and DAP can be a challenge for their complete visualization with micro-CT [32].

Berkhoff et al. [8] showed that CH was more effectively removed compared to the TAP regardless of the used irrigation techniques. They have attributed this result to the high diffusion capacity of TAP into the dentinal tubules. On the contrary, Eymirli et al. demonstrated that the remaining CH in the root canal system was significantly higher than TAP with a laser-activated system and they claimed that the small particle size of CH which leads to direct penetration of CH into the dentinal tubules [33]. In this study, two routinely used intracanal medicaments, CH and DAP, could not be removed entirely from the root canal space, and there was no statistically significant difference among those medicaments regarding retrievability with SNI, PUI, EDDY, and SWEEPS systems. This result is consistent with some studies in the literature that compare the removal of calcium and antibiotic pastes from the root canal system [6, 34]. Divergent results in the literature can be explained by the different irrigation activation systems, tooth morphologies, irrigation solutions and protocols, and evaluation methods.

EDDY and PUI have significantly increased the retrievability of intracanal medicaments from root canals compared to SNI; however, their superiority over each other has not been exhibited in this study in line with other studies in the literature [10, 14]. The higher velocity of irrigation solution created by PUI with enhanced fresh irrigant replacement could explain the larger amount of the removed intracanal medicaments [35]. Furthermore, improved irrigant fluid flow through higher frequency of EDDY system also leads to higher intracanal medicament removal from the root canal system [10]. Additionally, EDDY creates three-dimensional movement that triggers cavitation and acoustic streaming similar to the PUI, and therefore, similar efficiency of these irrigation activation systems might have been observed in terms of medicament removal.

Although the efficiency of SWEEPS technology regarding calcium hydroxide removal has been demonstrated in different clinical scenarios in the literature, there is a lack of information on its effectiveness in RETs. Kırmızı et al. [18] showed that although SWEEPS significantly increased the calcium hydroxide removal from the resorption cavities compared to the sonic and ultrasonic irrigation activation systems, there was no significant difference between the SWEEPS and PIPS groups. Moreover, Yang et al. [19] also found that the remnants of the calcium hydroxide were lesser in SWEEPS and PIPS groups than ultrasonic activation system, especially in the cervical third of the root canal system. According to the present findings, although complete removal could not be achieved with any of the irrigation activation systems, SWEEPS significantly improved the removal of CH and DAP in all experimental groups. This result can be explained by the unique activation mechanism of SWEEPS. It creates a sudden expansion of the second bubble produced by the second laser pulse, causing the primary bubble to collapse violently by applying additional pressure to the first bubble. This powerful shock wave generated by the secondary cavitation bubbles can be emitted in all root canal surfaces during the irrigation activation procedure. Accordingly, it can constitute the shear stress and vertical flows that can remove intracanal medicaments as well as debris, the smear layer, and biofilm effectively from the root canal surface [16, 17].

This study has some limitations that should be addressed. The vehicle used to mix the CH powder is an essential factor influencing the removal rate of CH from the root canal walls. Distilled water was selected as a vehicle in this study; however, if glycerin had been used, the removal of CH could have been more challenging [36]. In addition, the loss of medicament may have been occurred during the sectioning process of teeth. Moreover, irrigation activation systems may lead to structural changes and weakening of dentin when they are used in combination with EDTA [37]. Finally, since this study is the first study that evaluate the intracanal medicament efficiency of SWEEPS technology, removal amounts of medicaments have not been investigated regarding different parts of the teeth. This may lead to a lack of information, especially in the coronal part of the root, where the bond strength of barrier materials is important. Future detailed studies are needed to explore the efficiencies of these irrigation methods in terms of intracanal medicament removal and dentin erosion and/or demineralization in regenerative endodontics.

## Conclusion

The complete removal of intracanal medicaments from root canal space in RETs poses a risk. SWEEPS technology removed significantly higher amounts of CH and DAP compared to the other activation systems whereas SNI was the less effective system. PUI and EDDY showed similar effectiveness in terms of the ability to remove medicaments from root canal walls.

## Data Availability

The data that support the findings of this study are available from the corresponding author upon reasonable request.
